# Evaluation of Hypoglycemic Polyphenolic Compounds in Blueberry Extract: Functional Effects and Mechanisms

**DOI:** 10.3390/antiox13121490

**Published:** 2024-12-06

**Authors:** Yue Qin, Jielong Guo, Yuchen Lin, Yilin You, Weidong Huang, Jicheng Zhan

**Affiliations:** College of Food Science and Nutritional Engineering, China Agricultural University, Beijing 100083, China; qinyue@cau.edu.cn (Y.Q.); guojielong@tsinghua.edu.cn (J.G.); s20233061201@cau.edu.cn (Y.L.); yilinyou@cau.edu.cn (Y.Y.); weidonghuang@cau.edu.cn (W.H.)

**Keywords:** type 2 diabetes (T2D), blueberry polyphenol, insulin resistance, glycogenolysis, hypoglycemic effect

## Abstract

Blueberries are rich in polyphenols, which exhibit significant anti-diabetic activity. In this study, polyphenolic compounds with potential hypoglycemic activity were identified from blueberry polyphenol extract (BPE). This research focused on assessing the hypoglycemic effects of BPE and its polyphenolic compounds (dihydroquercetin and gallic acid) on diabetic mice induced by streptozotocin (STZ) and high-fat diet (HFD), as well as the related fundamental mechanisms. The findings revealed that BPE treatment effectively reduced levels of fasting blood glucose (FBG) by decreasing hepatic oxidative stress, regulating lipid metabolism disorders and improving insulin resistance. Investigations into the insulin signaling pathway revealed that BPE can modulate the expression of *Egfr*, *Insr*, *Irs-1*, *Pi3k* and *Akt*, thereby influencing glucose metabolism. This study provides a research foundation for considering blueberry polyphenols as a nutritional dietary supplement for the prevention and intervention of diabetes.

## 1. Introduction

In 2021, according to estimates, 537 million individuals are affected by diabetes, which represents 10.5% of the adult population globally. The incidence of diabetes is expected to increase 643 million (11.3%) by the year 2030, with projections soaring to 783 million (12.2%) by 2045 [1]. Diabetes can be categorized into type 1 diabetes, type 2 diabetes (T2D), gestational diabetes and several particular kinds of diabetes [2]. Among these, the prevalence of T2D (a metabolic disease) is the highest, constituting over 90% of diagnosed cases of diabetes [3]. T2D is a metabolic disorder with persistent high blood glucose levels. Insulin resistance (IR) is a hallmark feature of T2D. If the body remains in a prolonged state of hyperglycemia without intervention, it may lead to damage in various organs, resulting in multiple diabetes-related complications, including diabetic ketoacidosis, diabetic retinopathy, diabetic nephropathy, diabetic neuropathy and cardiovascular disorders [4,5,6]. Over the past few decades, a range of antihyperglycemic agents have been utilized in the treatment of T2D, including metformin; sulfonylureas, such as glipizide; alpha-glucosidase inhibitors, such as acarbose; and glucagon-like peptide-1 receptor (GLP-1R) agonists [7,8]. However, these agents can cause a variety of side effects, including skin allergies, adverse gastrointestinal reactions, hepatic and renal damage and cardiovascular risks [8,9,10,11]. Therefore, identifying safe and effective hypoglycemic agents or functional foods derived from natural sources is of paramount significance. Although natural compounds are often regarded as safer alternatives, it is important to recognize that natural extracts may also induce adverse effects depending on factors such as dosage and duration of exposure. Furthermore, toxic substances can be found in nature, and their safety profiles should be thoroughly evaluated before use.

In recent years, natural functional ingredients derived from food and plants have been extensively utilized in the prevention and management of metabolic disorders, leading to notable interest among both researchers and the general public. The Food and Agriculture Organization of the United Nations has identified blueberries (*Vaccinium* spp.) as one of the five fruits beneficial for health, due to their high significant biological activity attributable to the rich presence of polyphenolic compounds, which are noted for their characteristics, including antioxidant effects, anti-cancer characteristics and anti-inflammatory activities [12,13,14,15]. Evidence suggests a negative correlation between the consumption of polyphenols and the risk of T2D [16]. Our previous research indicated that blueberry extract can enhance glucose tolerance and insulin sensitivity in diet-induced obese mice and alleviate obesity-related metabolic syndrome by modulating plasma bile acids through gut microbiota [17]. The consumption of fresh blueberries can enhance postprandial blood glucose levels in sedentary subjects [18]. Randomized clinical trials indicated that supplementation with blueberry extract or freeze-dried blueberries is beneficial for glycemic control and hemoglobin A1c (HbA1c) levels in subjects with T2D [19,20]. However, the mechanisms involved require further investigation.

To evaluate the impacts of blueberry polyphenols on T2D, this study employed an HFD-combined-with-STZ-induced mice diabetes model. We examined the improvement in glucose and lipid metabolism in diabetic mice from blueberry polyphenol extract (BPE) and their key active polyphenolic components. The findings could provide valuable insights into the possibility of blueberry polyphenols as a dietary health supplement.

## 2. Materials and Methods

### 2.1. Materials and Chemicals

BPE was acquired from Jiangsu Wotian Biotechnology Co., Ltd. (Lianyungang, China). STZ (#S0130) and palmitic acid (#P0500) were purchased from Sigma Aldrich (St. Louis, MO, USA). Citric acid/citrate buffer solution (#C1013) (0.1 M pH 4.5) was purchased from Beijing Solarbio Science & Technology Co., Ltd. (Beijing, China). Dihydromyricetin and gallic acid were purchased from Yuanye Bio-Technology (Shanghai, China) for animal experiments and from Target Molecule Corp. (Boston, MA, USA) for cell experiments. Metformin hydrochloride was purchased from TCI (Tokyo, Japan) for animal experiments and from Target Molecule Corp. (Boston, MA, USA) for cell experiments. Quercetin, rutin, chlorogenic acid, gentisic acid, syringic acid, methyl gallate and pyrogallol were purchased from Target Molecule Corp. (Boston, MA, USA). Catechol was purchased from Chengdu MUST bio-technology Co., Ltd. (Chengdu, China). Dulbecco’s modified Eagle’s medium (DMEM) was purchased from Biosharp (Hefei, China). Fetal bovine serum was purchased from Biological Industries (Haemek, Israel) and trypsin was obtained from Gibco (Grand Island, NY, USA). Insulin was obtained from Novo Nordisk (Copenhagen, Denmark). Antibodies against p-PI3K (#4228), PI3K (#4257), p-AKT (#4691) and AKT (#4060) were obtained from Cell Signaling Technology (Danvers, MA, USA).

### 2.2. Analysis of Polyphenol

Total polyphenol content in BPE was assessed using a modified Folin–Ciocalteu method [17]. Total flavonoid content was assessed using a previous spectrophotometry method [21]. Total anthocyanin content was assessed using a pH differential method [17]. The results were presented as gallic acid, rutin or cyanidin-3-glucoside equivalent of BPE (mg/g), respectively.

The analysis of compounds in BPE was conducted using UHPLC-MS/MS. The specific methodology is as follows: separation was performed on an ACQUITY UPLC HSS T3 chromatographic column (2.1 × 100 mm, 1.8 μm, Waters Corp, Milford, MA, USA) with a column temperature of 35 °C and a flow rate of 0.2 mL/min. The mobile phase for the chromatographic column consisted of solvent A (0.1% formic acid, deionized water) and solvent B (0.1% formic acid, acetonitrile). The separation of compounds was conducted under the following gradient conditions: 0–10 min, 0–30% B; 10–25 min, 30–40% B; 25–40 min, 40–70% B; 40–45 min, 70–100% B; 45–60 min, 100% B; 60–60.5 min, 100–0% B; 60.5–70 min, 100% A, with an injection volume of 10 μL. MS analysis was performed using a Q Exactive Orbitrap high-resolution mass spectrometer (Thermo Fisher Scientific, Waltham, MA, USA) with a detection mode of Full MS-ddMS2. The full MS scan ranges were set from 100–1200 m/z. The MS1 resolution was set to 70,000, and the MS2 resolution to 17,500. The ion source voltage was maintained at 3.2 kV, with a capillary temperature of 320 °C, an auxiliary gas heater temperature of 350 °C, a sheath gas flow rate of 40 L/min and an auxiliary gas flow rate of 15 L/min. Data analysis was carried out using Compound Discoverer 3.3 software, employing both a local database and the mzCloud online database for the structural identification of the compounds.

### 2.3. Cell Culture and Treatments

The HepG2 cells were kindly provided by Prof. Hongbo Hu from China Agricultural University in Beijing, China. HepG2 cells were cultured in DMEM medium supplemented with 10% fetal bovine serum at 37 °C and 5% CO_2_.

As described below, the IR model was induced in HepG2 cells [22]. HepG2 cells were induced using DMEM complete medium containing 200 μM palmitic acid (with 1% BSA), and simultaneously treated with different polyphenols for 24 h, with the following groups: normal control (NC group); IR model control (MC group); 2 mM Metformin positive control (PC group); 10 μg/mL blueberry polyphenol extract (B group); 10 μM dihydromyricetin (D group); 10 μM gallic acid (G group).

### 2.4. MTT Assay

The MTT assay was employed to evaluate the viability of HepG2 cells. HepG2 cells were plated in 96-well plates, and treatments were applied once the cells reached 40–60% confluence, with negative and blank control groups (without cells) included. The assay was conducted according to the instructions (#C0009S, Beyotime Institute of Biotechnology, Shanghai, China). After 24 h of treatment of different concentrations of compounds (0, 10, 25, 50, 75, 100, 200, 400 μM), the medium was discarded followed by the addition of 100 μL of 0.5 mg/mL MTT solution to each well. After incubating for 4 h at 37 °C, each well received 100 μL of formazan solution and was incubated for a suitable duration to ensure the complete dissolution of the crystals. The absorbance was measured at 570 nm for each well.

### 2.5. Glucose Consumption Assay

HepG2 cells were seeded in 96-well plates (1 × 10^4^ cells/well) and subjected to different polyphenol administrations. Blueberry extract was applied at 10 mg/L, and polyphenol compounds at 10 μM, for 24 h. After treatment and two washes with PBS, 200 μL of serum-free, sodium pyruvate-free DMEM containing 10⁻⁶ mol/L insulin was added to each well for a 2 h incubation. The blank wells were set up with only medium, without any cells. Glucose levels in the medium were determined in accordance with the instructions supplied by the glucose assay kit (#A154-1-1, Nanjing Jiancheng Bioengineering Institute, Nanjing, China). The calculation of glucose consumption was performed as follows: glucose concentration in the blank well medium—glucose concentration in the sample well medium.

### 2.6. Glycogen, Hexokinase (HK), Pyruvate Kinase (PK), Glucose-6-Phosphatase (G6P)

After treatment for 24 h by polyphenols of 10 μM (and blueberry extract of 10 mg/L), the cells were transferred to a 1.5 mL centrifuge tube and centrifuged at 1000 rpm for 2 min at 4 °C, followed by two washes with PBS. An appropriate amount of extraction buffer was added, and the cells were disrupted; measurements were performed according to the kit’s instructions (HK#BC0745, PK#BC0545, G6P#BC3325, Beijing Solarbio Science & Technology Co., Ltd., Beijing, China). Protein quantification was performed using the BCA method, following the instructions of the kit (#P0009, Beyotime Institute of Biotechnology, Shanghai, China).

### 2.7. Animal Experiment Design

**Figure 1:**
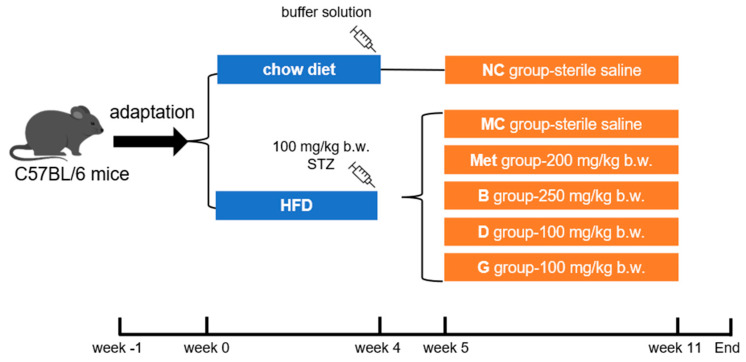
Feeding and supplementation protocol. HFD, high fat diet; NC group, normal control; MC group, model control; Met group, metformin positive medicinal control; B group, blueberry polyphenol extract; D group, dihydromyricetin; G group, gallic acid.

The male C57BL/6 mice (5 weeks old, 18–20 g) were purchased from Beijing Vital River Laboratory Animal Technology Co., Ltd. (Beijing, China). The mice were caged under standard conditions (12 h/12 h light–dark cycle, humidity at 50 ± 10%, temperature 22 ± 2 °C). Food and water were freely accessed. After adaptive feeding for 1 week, mice were randomly selected as the normal control group (NC group), fed a chow diet (Beijing Keao Xieli Feed Co., Ltd., Beijing, China). The remaining mice were provided with an HFD (#D12492, Research Diets, New Brunswick, NJ, USA). After 4 weeks, mice fed the HFD were intraperitoneally injected with STZ (100 mg/kg b.w., dissolved in a 0.1 M pH 4.5 citric acid/citrate buffer solution). After 1 week, fasting blood glucose (FBG) was measured, and mice with FBG higher than 11.1 mmol/L were considered as diabetic. Diabetic mice were randomly divided into 5 groups according to FBG level: model control (MC group), metformin positive medicinal control group (Met)—200 mg/kg b.w., blueberry polyphenol extract (B group) group—250 mg/kg b.w., dihydromyricetin (D group) group—100 mg/kg b.w. and gallic acid (G group) group—100 mg/kg b.w. The NC group and MC group were also given sterile saline. The mice were orally gavaged every day for 6 weeks. In the end, the liver, pancreas and serum of the mice were collected for further experiments. This process is shown in Figure 1. 

### 2.8. OGTT and IPITT

During the 6th week, the oral glucose tolerance test (OGTT) and intraperitoneal insulin tolerance test (IPITT) were performed after overnight fasting. The mice were treated with 1.5 g/kg glucose solution by oral gavage for OGTT and 1 U/kg insulin by intraperitoneal injection for IPITT. Blood glucose levels of OGTT were measured at 0, 15, 30, 60, 90 and 120 min. Blood glucose levels of IPITT were measured at 0, 15, 30, 45 and 60 min. The area under the curve (AUC) was used to visualize the data obtained from the OGTT and IPITT.

### 2.9. Serum Biochemical Indexes

After an 8 h fast, the FBG levels were determined by blood glucose meter (ACCUCHEK) and its corresponding test strips.

Serum insulin levels and glycated serum protein (GSP) were assayed using ELISA kits (Borui Changyuan Technology Co., Ltd., Beijing, China). Total cholesterol (TC, #A111-1-1), triglyceride (TG, #A110-1-1), high-density lipoprotein cholesterol (HDL-C, #A112-1-1), low-density lipoprotein cholesterol (LDL-C, #A113-1-1), alanine aminotransferase (ALT, #C010-2-1) and aspartate aminotransferase (AST, #C009-2-1) were assayed using commercial kits (Nanjing Jiancheng Bioengineering Institute, Nanjing, China).

The HOMA-IR (homeostasis model assessment of insulin resistance) was calculated with the following equation:

HOMA-IR = [fasting glucose (mmol/L) × fasting insulin (mU/L)]/22.5

### 2.10. Oxidative Stress in Liver

Oxidative stress in liver was analyzed through the measurement of levels of malondialdehyde (MDA, #BC0025), superoxide dismutase (SOD, #BC5165) and catalase (CAT, #BC0205), which were measured using commercial kits (Beijing Solarbio Science & Technology Co., Ltd., Beijing, China).

### 2.11. Histopathological Observation of Liver

Histopathological observation of mouse liver tissues was conducted using H&E, Oil Red O and PAS staining techniques. Liver tissue samples were immersed in 4% paraformaldehyde-buffered solution, followed by embedding in paraffin and sectioning into thin slices for H&E and PAS staining. Frozen sections of the OCT-embedded samples were made for Oil Red O staining. Observations and photography were performed under a microscope. Finally, the images were examined and assessed for pathological change analysis.

### 2.12. Analyses of mRNA Expression

The RNA isolator Total RNA Extraction Reagent (#R401-01, Vazyme Biotech Co., Ltd., Nanjing, China) was used to extract total RNA from the tissues of mice. The obtained RNA was reverse transcribed into cDNA using a Super plus qPCR RT kit with gDNA remover (#MF166-plus, Mei5 Biotechnology Co., Ltd, Beijing, China). Real-time fluorescence quantitative PCR (qPCR) was performed using HiPer SYBR Premix EsTaq (#MF787, Mei5 Biotechnology Co., Ltd, Beijing, China). The results were calculated by 2^−ΔΔct^. Primers were synthesized by Sangon Biotech (Shanghai, China) Co., Ltd. Table S1 displays the primer sequences.

### 2.13. Western Blot Assay

The tissue was lysed in cold RIPA lysis buffer (#P0013B, Beyotime Biotechnology, Shanghai, China) with PMSF (#ST506, Beyotime Biotechnology, Shanghai, China) and protein phosphatase inhibitor (#P1260, Beijing Solarbio Science & Technology Co., Ltd., Beijing, China). After centrifugation at 12,000× *g* for 15 min at 4 °C, the protein quantification was performed using the BCA method, following the instructions of the kit (#P0009, Beyotime Institute of Biotechnology, Shanghai, China). Protein was prepared for denaturation at 95 °C for 8 min. Then, 40 μg proteins was added and was separated with 10% SDS-PAGE and transferred to PVDF membranes. After blotting with blocking buffer (#P0252, Beyotime Biotechnology, Shanghai, China) for 10 min at room temperature, the membranes were incubated overnight at 4 °C with primary antibodies. After three washes with wash buffer, the membranes were incubated with secondary antibodies conjugated to horseradish peroxidase for 1 h at room temperature. The analysis was conducted using enhanced BeyoECL reagents (Beyotime Biotechnology, Shanghai, China).

### 2.14. Statistics

All data are expressed as mean ± standard error of mean (SEM) and analyzed using GraphPad Prism 8 (La Jolla, CA, USA). All data sets were tested for normality using the Shapiro–Wilk test, and then significant differences between groups were examined using one-way ANOVA for repeated measures, followed by Dunnett’s test. Lowercase letters indicate statistical differences. The *p* value < 0.05 was deemed statistically significant. * *p* < 0.05, ** *p* < 0.01, *** *p* < 0.001, **** *p* < 0.0001 vs. group MC.

## 3. Results

### 3.1. Quantitation of Polyphenols in BPE

As shown in Table 1, BPE contains 635.58 mg/g total polyphenols, 118.97 mg/g total flavonoids and 246.13 mg/g total anthocyanins.

In total, 57 polyphenolic compounds were identified, as shown in Table S2. Based on previous literature, two highly bioactive polyphenol compounds (dihydromyricetin and gallic acid) were selected for further validation of hypoglycemic activity.

### 3.2. In Vitro Evaluation of BPE Polyphenols Exhibiting Hypoglycemic Activity

Ten polyphenolic compounds were selected for in vitro evaluation of hypoglycemic active components (Table 2). The in vitro IR model in HepG2 cells was induced using palmitic acid. To evaluate the cytotoxicity of blueberry polyphenols, cell viability was measured in HepG2 cells using the MTT assay. As illustrated in Figure S1, 10 μM of polyphenol monomers and 10 mg/L of blueberry extract did not significantly affect the growth of HepG2 cells; therefore, further experiments were conducted at these concentrations.

**Figure 2:**
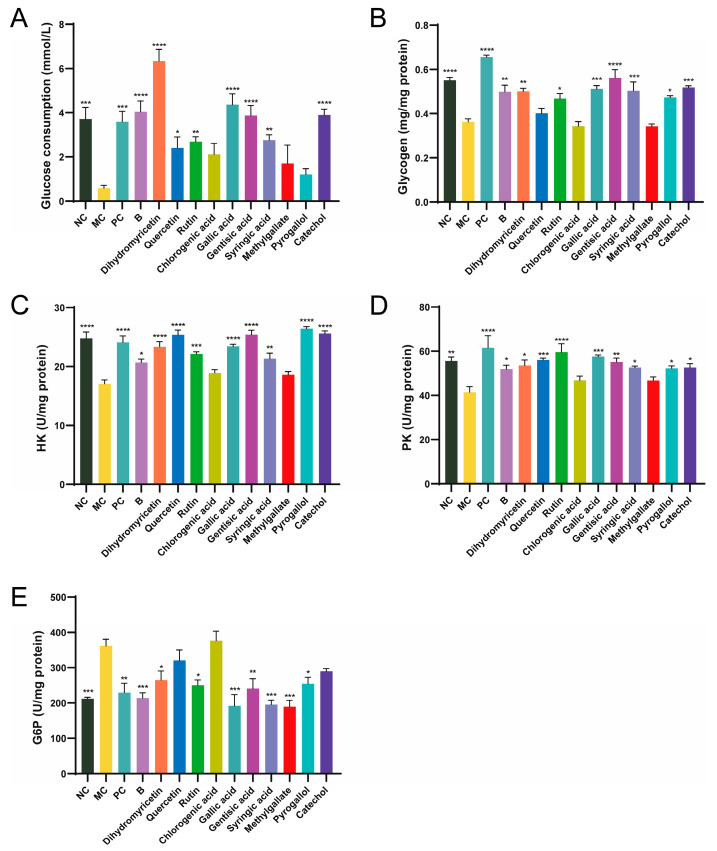
Effects of blueberry polyphenol in IR HepG2 cells. Blueberry extract was treated at 10 mg/L, and polyphenol compounds at 10 μM for 24 h. (**A**) Glucose consumption; (**B**) Glucose content; (**C**–**E**) Activity of HK, PK and G6P (n = 4). Data are expressed as the mean ± SEM. * *p* < 0.05 versus MC group; ** *p* < 0.01 versus MC group; *** *p* < 0.001 versus MC group; **** *p* < 0.0001 versus MC group.

Glucose consumption reflects the ability of cells to uptake glucose. Compared to the NC group, glucose consumption in the MC group was significantly reduced, decreasing by 84.32%, indicating successful establishment of the insulin resistance model (Figure 2A). The positive control metformin (PC group) enhanced glucose uptake in cells, probably resulting from enhanced insulin resistance in the cells and the modulation of glucose metabolism. After 24 h of BPE treatment, glucose consumption returned to normal levels, suggesting that insulin resistance had been alleviated and improved. Among the ten polyphenol monomers, dihydromyricetin exhibited remarkable regulatory ability, not only restoring glucose uptake levels in IR cells but also significantly promoting glucose uptake, effectively reversing the state of insulin resistance. Quercetin, rutin, gallic acid, gentisic acid, syringic acid and catechol can improve glucose uptake levels in IR cells to varying degrees.

Insulin resistance results in decreased glucose utilization by the cells, along with an inhibition of glycogen synthesis within them [23]. In comparison to the NC group, glycogen content in the MC group decreased by 30.46% (Figure 2B). The PC group also showed an increase in glycogen accumulation in IR cells. After 24 h of BPE treatment, the glycogen levels of HepG2 cells were significantly improved, and dihydromyricetin, rutin, gallic acid, gentisic acid, syringic acid, pyrogallol and catechol improved glycogen levels in IR cells to varying degrees.

HK, PK and G6P are three key enzymes involved in glucose metabolism, which are crucial for the cellular utilization or synthesis of glucose. Compared to the NC group, the activity of HK and PK in IR-HepG2 cells (MC group) was significantly reduced, while G6P was significantly elevated, indicating that the activities of these three enzymes underwent significant changes under insulin resistance (Figure 2C–E). BPE and the ten polyphenol monomers can improve the activities of the three enzymes to varying degrees. Interestingly, dihydromyricetin significantly boosted the ability of cells to utilize glucose, whereas gallic acid consistently demonstrated excellent results across multiple validations. According to ADMET (absorption, distribution, metabolism, excretion and toxicity) prediction, dihydromyricetin and gallic acid both exhibit good water solubility and high intestinal absorption rates in humans, but are poorly distributed to the brain (Table S3). Neither compound shows hepatotoxicity. Taking various factors into account, this study selected dihydromyricetin and gallic acid as representative polyphenol monomers for further investigation.

### 3.3. Effects of BPE on Blood Glucose Levels in Diabetic Mice

**Figure 3:**
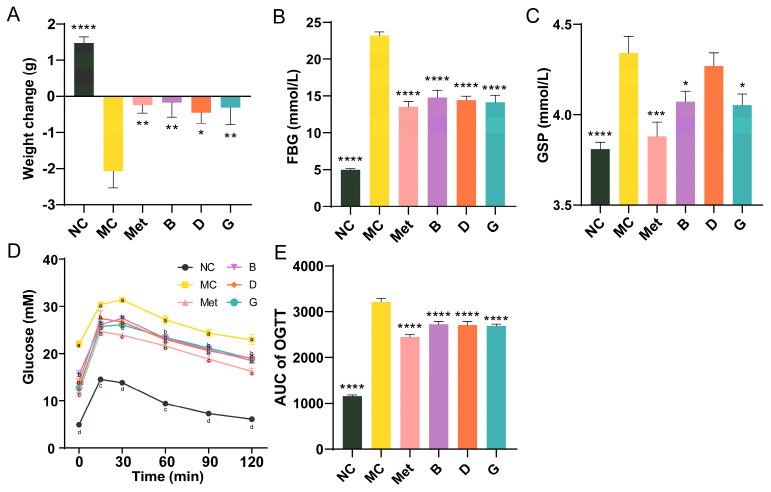
Effects of BPE on blood glucose levels in diabetic mice. (**A**) Weight change of mice after 6 weeks of treatment; (**B**) FBG; (**C**) GSP; (**D**) and (**E**) OGTT results of the mice (n = 6−8). Data are expressed as the mean ± SEM. * *p* < 0.05 versus MC group; ** *p* < 0.01 versus MC group; *** *p* < 0.001 versus MC group; **** *p* < 0.0001 versus MC group. The values with different letters (a, b, c or d) are significantly different (*p* < 0.05) between each group at the same time.

To investigate the effects of blueberry polyphenols on diabetic mice, a T2D mice model was established using C57BL/6 mice injected intraperitoneally with STZ after 4 weeks of HFD. After 6 weeks of treatment, the body weight of mice in the NC group showed normal growth, indicating that the mice were in good health (Figure 3A). In contrast, the body weight of mice in the MC group significantly decreased. Treatment with the positive drug and the three polyphenols effectively prevented weight loss and alleviated the detrimental effects of diabetes on the overall health of the mice. FBG is the most rapid and convenient method for evaluating blood glucose levels in individuals with diabetes. Our research suggested that the MC group of mice exhibited significantly higher FBG levels than the NC group, suggesting that the diabetic mouse model is developed. After 6 weeks of intervention, the FBG of mice in groups B, D and G was significantly improved and reduced to the level of Met group (Figure 3B). GSP reflects the blood glucose concentrations from the past 2 to 3 weeks. Compared with MC group, the GSP of mice in the Met, B and G groups was significantly improved, which was consistent with the trend of FBG (Figure 3C). While the GSP of mice in the D group did not significantly differ from that of the MC group, a tendency for decline was still noted. The results of the glucose tolerance test (OGTT) in mice showed that the blood glucose of mice in the NC group peaked at 15 min, then gradually decreased and converged to the baseline level at 2 h (Figure 3D). The mice in the MC group had an initial fasting glucose higher than 20 mmol/L and were in a state of sustained hyperglycemia, and the AUC was notably elevated in comparison to the NC group, which indicated that the glucose tolerance of MC group was significantly impaired (Figure 3E). The four intervention groups showed significant improvement in initial blood glucose, with AUC significantly lower than MC group. The Met group exhibited the most favorable results.

### 3.4. Effect of BPE on Insulin Resistance in Diabetic Mice

**Figure 4:**
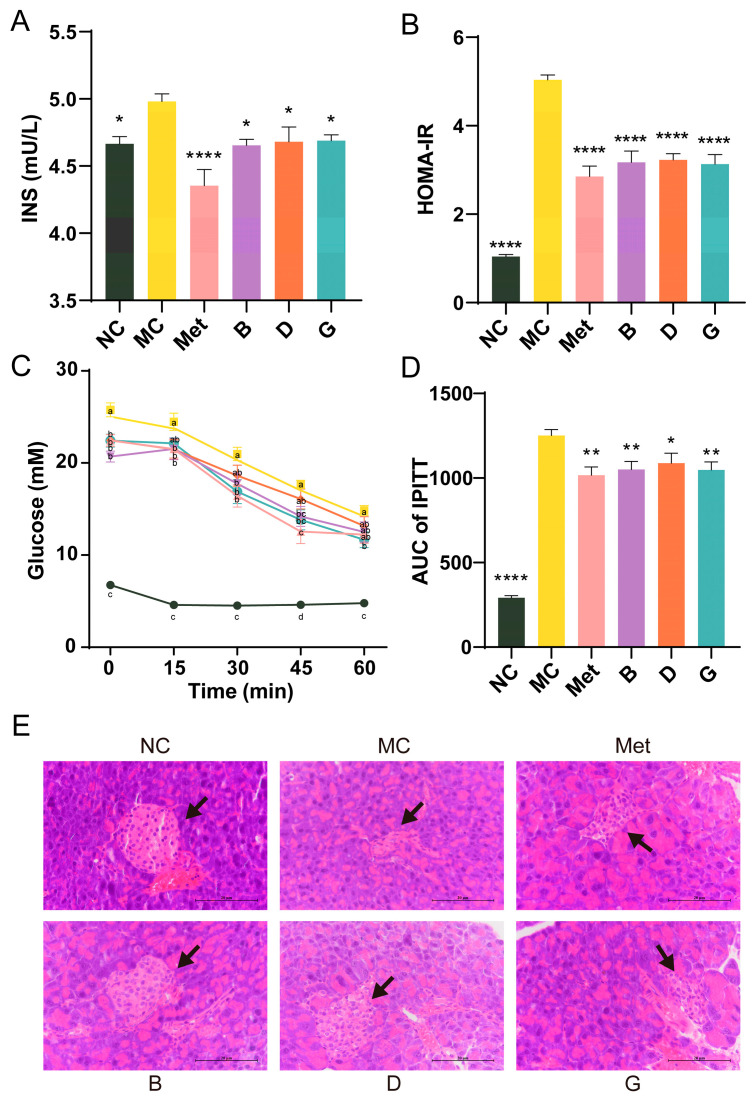
Effects of BPE on insulin resistance in diabetic mice. (**A**) Serum insulin level; (**B**) HOMA-IR; (**C**,**D**) IPITT results of the mice (n = 6–8); (**E**) H&E staining of pancreas. (400× magnification, scale: 20 μm). The black arrow indicates the islets. Data are expressed as the mean ± SEM. * *p* < 0.05 versus MC group; ** *p* < 0.01 versus MC group; **** *p* < 0.0001 versus MC group. The values with different letters (a, b, c, d) are significantly different (*p* < 0.05) between each group.

The serum insulin levels in the MC group exhibited a significant increase, and the homeostasis model assessment of insulin resistance (HOMA-IR) of the MC group was 4.82 times elevated compared to the NC group, suggesting that the mice exhibited severe insulin resistance (Figure 4A,B). After treatment with BPE and polyphenol compounds (B, D, G groups), serum insulin levels were significantly reduced, resulting in a substantial alleviation of the insulin resistance state, approaching the levels observed in the Met group. The IPITT experiment confirmed that the initial FBG levels in the MC group were significantly elevated, consistent with previous experimental results (Figure 4C). In comparison to the NC group, the AUC of the MC group was notably elevated, reflecting decreased insulin sensitivity (Figure 4D). Treatment with polyphenols (B, D, G groups) resulted in significant improvements in insulin sensitivity, with no significant difference compared to the Met group. These findings indicated the potential benefits of BPE in improving insulin sensitivity and reducing insulin resistance in T2D mice. Additionally, Figure 4E shows that the NC group exhibited intact pancreatic islet structures, with regular elliptical shape, evenly distributed cells and clear nuclei. Conversely, the diabetic mice in the MC group showed pancreatic islet atrophy, irregular morphology and disrupted cell clusters, alongside a reduction in islet cell quantity. Treatment with polyphenols (B, D, G groups) improved the pancreatic islet morphology and increased islet cell count, partially restoring the pancreatic damage.

### 3.5. Effects of BPE on Blood Lipid Levels in Diabetic Mice

**Figure 5:**
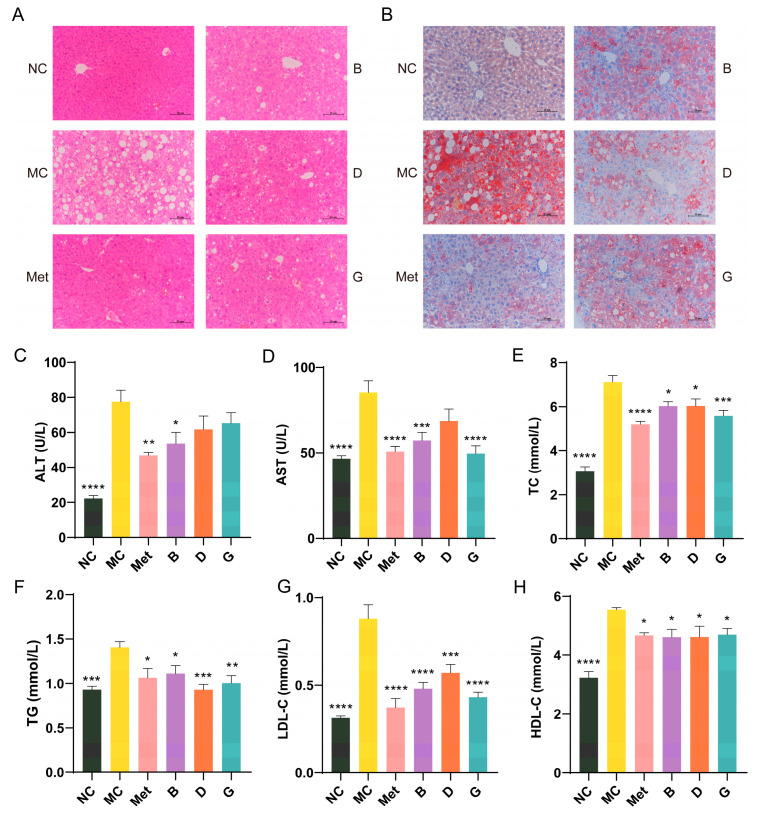
Effects of BPE on blood lipid levels in diabetic mice. (**A**) H&E staining of liver. (200× magnification, scale: 20 μm); (**B**) Oil red O staining of liver. (200× magnification, scale: 20 μm); (**C**,**D**) Serum ALT and AST level; (**E**–**H**) serum TC, TG, LDL-C and HDL-C level (n = 6–8). Data are expressed as the mean ± SEM. * *p* < 0.05 versus MC group; ** *p* < 0.01 versus MC group; *** *p* < 0.001 versus MC group; **** *p* < 0.0001 versus MC group.

Lipid metabolic disorders are often present alongside diabetes [24]. Figure 5A shows the histological morphology of the mouse liver. In comparison to the NC group, the hepatocytes in the MC group showed disorganized arrangement, with disrupted hepatic cord structures and substantial vacuolation in the cytoplasm of hepatocytes, resulting in cellular hypertrophy. The nuclei of the hepatocytes were compressed and displaced, leading to pronounced steatosis in the liver. After treatment with BPE (groups B, D, G), although the effects were not as pronounced as those observed in the Met group, the disorder in the arrangement of the hepatic cords showed varying degrees of restoration, and the vacuoles decreased in size, suggesting a return to normal architecture. Oil Red O staining indicated that the liver in the MC group experienced significant lipid deposition; however, following positive drug and polyphenol treatments, this lipid accumulation was markedly improved, with a noticeable reduction in the size of the lipid droplets, indicating that the liver was partially protected (Figure 5B).

ALT and AST are crucial biomarkers for assessing liver function, indicating the extent of hepatocellular damage. When liver damage occurs, AST and ALT are released in large quantities into the blood. The MC group of mice exhibited significantly increased serum levels of AST and ALT in comparison to the NC group, suggesting hepatic injury in the diabetic mice (Figure 5C,D). Treatment with BPE (groups B, D, G) demonstrated protective effects on the liver. Dyslipidemia is a significant factor in T2D. To evaluate the lipid-lowering effect of BPE on diabetic mice, levels of TG, TC, LDL-C and HDL-C were measured (Figure 5E–H). The TG, TC and LDL-C levels in the MC group were notably increased, indicating severe lipid metabolism abnormalities in the diabetic mice. Following treatment with BPE (groups B, D, G), these parameters showed significant improvement, suggesting that blueberry polyphenols can modulate lipid metabolic disorders in diabetic mice. HDL-C contributes to the clearance of LDL-C from the blood and its transport back to the liver for metabolism and excretion, thereby reducing the risk of cardiovascular diseases, which is often regarded as beneficial to health. However, excessively elevated HDL-C levels can also have adverse health effects. Investigations have indicated that the potential for stroke and mortality is heightened with both low and high HDL-C levels, exhibiting a U-shaped relationship [25,26]. Treatment with BPE (groups B, D, G) was able to restore HDL-C levels to the normal range.

### 3.6. Effect of BPE on the Level of Oxidative Stress in the Liver

**Figure 6:**
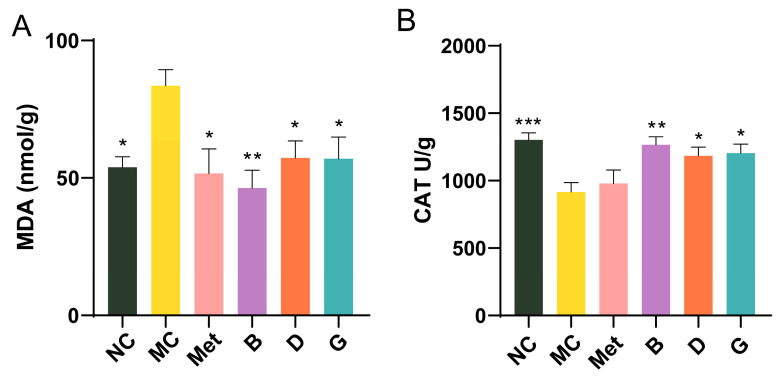
Effect of BPE on the level of oxidative stress in the liver. (**A**) Liver MDA level; (**B**) Liver CAT level (n = 6). Data are expressed as the mean ± SEM. * *p* < 0.05 versus MC group; ** *p* < 0.01 versus MC group; *** *p* < 0.001 versus MC group.

Oxidative stress is closely linked to the development of T2D. Malondialdehyde (MDA), a byproduct of lipid peroxidation, is commonly regarded as a biomarker for oxidative stress. Within the body’s metabolic processes, a substantial amount of reactive oxygen species (ROS) is generated, alongside the expression of antioxidant enzymes including catalase (CAT), which work to neutralize ROS and maintain cellular redox homeostasis. In the MC group, MDA levels were measured to be 1.55 times higher than those in the NC group, with CAT levels significantly reduced, reflecting severe oxidative stress in diabetic mice (Figure 6A,B). Intervention with BPE (groups B, D, G) significantly improved the MDA and CAT levels in the diabetic mice, suggesting that BPE may counteract the oxidative stress induced by hyperglycemia, thereby strengthening the organism’s resistance to oxidative damage.

### 3.7. The Effect of BPE on the Expression of Key Genes of Glucose Metabolism and Lipid Metabolism in Diabetic Mice

**Figure 7:**
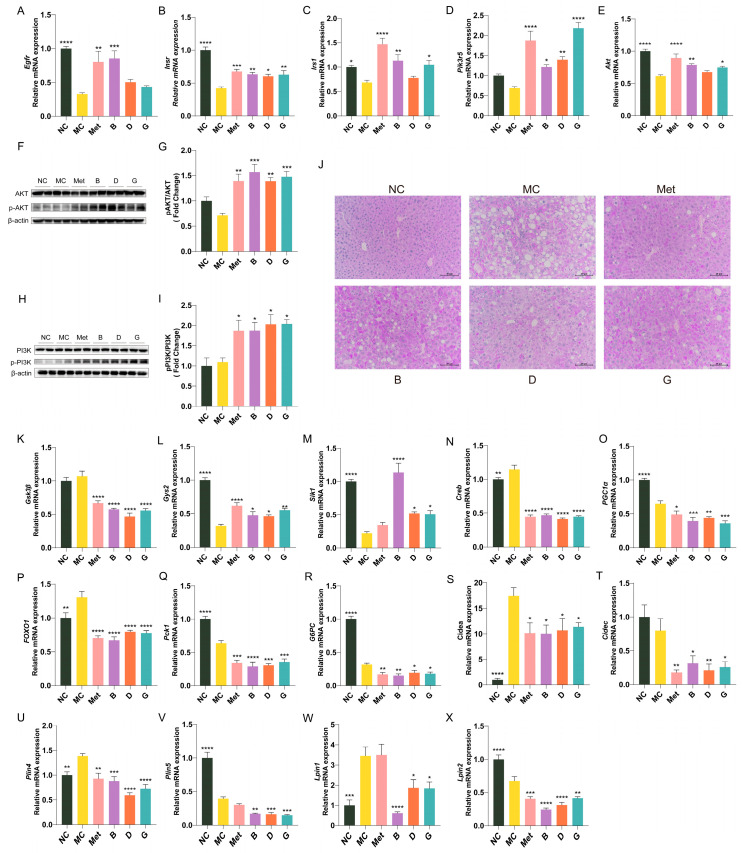
Effect of BPE on the expression of key genes of glucose metabolism and lipid metabolism in diabetic mice (n = 6). (**A**–**E**) *Egfr*, *Insr*, *Irs1*, *Pik3r5*, *Akt* mRNA expression; (**F**,**H**) Representative immunoblots of AKT, p-AKT, PI3K, p-PI3K in liver. (**G**,**I**) Quantification of protein levels of p-AKT/AKT and p-PI3K/PI3K (n = 4). (**J**) PAS staining of liver (200 × magnification, scale: 20 μm); (**K**–**X**) *Gys2*, *GSK3β*, *Sik1*, *Creb*, *PGC-1α*, *FOXO1*, *PEPCK*, *G6Pase*, *Cidea*, *Cidec*, *Plin4*, *Plin5*, *Lpin1*, *Lpin2* mRNA expression (n = 6). Data are expressed as the mean ± SEM. * *p* < 0.05 versus MC group; ** *p* < 0.01 versus MC group; *** *p* < 0.001 versus MC group; **** *p* < 0.0001 versus MC group.

The expression profiles of essential genes involved in the insulin signaling pathway in the liver are illustrated in Figure 7A–E. Compared to the NC group, the gene expression levels of *Egfr*, *Insr*, *Irs-1*, *Akt* and *Pi3k* in the liver of the MC group were notably decreased. This indicates a decrease in insulin receptors and restricted insulin signaling in diabetic mice, resulting in an inability to utilize endogenous insulin. The mRNA expression levels of *Egfr*, *Insr*, *Irs-1*, *Akt* and *Pi3k* in the polyphenol treatment groups showed varying degrees of improvement, indicating that these polyphenols may regulate the PI3K/Akt signaling pathway to mediate glucose metabolism. Notably, group B exhibited the most significant improvement; among the two polyphenol compounds, gallic acid (group G) demonstrated a greater ameliorative effect than dihydromyricetin (group D). In order to investigate the effects of blueberry polyphenols on the insulin signaling pathway, we confirmed the phosphorylation levels of PI3K and AKT by Western blot (Figure 7F–I). Compared with the MC group, the protein levels of p-PI3K/PI3K and p-AKT/AKT in mice liver treated with polyphenols were significantly increased, indicating that blueberry polyphenols can regulate insulin signal transduction.

Hepatic glycogen storage analysis was performed through PAS staining. Hepatic glycogen displayed a purple coloration, which darkened in intensity with increasing glycogen content. As depicted in Figure 7J, the quantity of hepatic glycogen in the MC group was markedly reduced, while metformin treatment appropriately restored hepatic glycogen levels to normal. Groups B, D and G demonstrated significantly enhanced glycogen levels. Figure 7K–R illustrate the expression profiles of key genes linked to glycogen synthesis and gluconeogenesis in the hepatic tissue of mice. In the MC group, the glycogen synthase 2 (*Gys2*) was significantly reduced, while the expression level of glycogen synthase kinase-3β (*GSK3β*), an inhibitor of glycogen synthesis, was elevated, indicating impaired glycogen synthesis in the MC group. After treatment with BPE and polyphenol compounds, there was a notable enhancement in the expression levels of two glycogen synthesis-related enzymes, suggesting that BPE may facilitate hepatic glycogen synthesis in T2D mice. As regulators of hepatic gluconeogenesis, salt-induced kinase 1 (*Sik1*), cAMP response element-binding protein (*Creb*), peroxisome proliferator-activated receptor gamma coactivator 1α (*PGC-1α*) and forkhead box O1 (*FOXO1*) collectively engage in the modulation of gluconeogenesis. The blueberry polyphenol treatment group was able to modulate the expression of upstream genes involved in gluconeogenesis, resulting in a reduction of the gene expression of essential enzymes in gluconeogenic pathway, namely *PEPCK* and *G6Pase*, thereby inhibiting gluconeogenesis.

Figure 7S–X illustrate the expression profile of key genes involved in lipid metabolism in mouse liver. Compared to the MC group, intervention with blueberry polyphenols resulted in varying degrees of decreased expression of the cell death-inducing DNA fragmentation factor-alpha-like effector a (*Cidea*), *Cidec*, Perilipin 4 (*Plin4*), Perilipin 5 (*Plin5*), lipin 1 (*Lpin1*) and lipin (*Lpin2*) genes in the livers of mice, indicating an improvement in hepatic lipid droplet formation and metabolic homeostasis in T2D mice.

## 4. Discussion

As the economy evolves and lifestyle changes, the rate of diabetes occurrence is escalating at a worrisome speed. Diabetes has emerged as a significant public health concern in modern society, attracting substantial attention from researchers and healthcare professionals. For T2D patients, treatment necessitates a multifaceted approach that incorporates a combination of pharmacological interventions, dietary modifications, exercise and lifestyle changes. Blueberries, recognized as a health-promoting fruit rich in polyphenolic compounds, have demonstrated potential glycemic-lowering effects in previous studies. Ethanol extracts of blueberries have been demonstrated to enhance glucose tolerance in obese mice induced with metabolic syndrome by an enriched sucrose diet diet [27]. Prolonged consumption of blueberry juice caused a significant decline in postprandial blood glucose levels and better glucose tolerance and insulin sensitivity in prediabetic rats [28]. Blueberry extracts may exert their hypoglycemic activity by stimulating the expression of GLUT-2 and PPARγ in normal hepatic LO2 cells [29]. This study investigates the hypoglycemic effects of blueberry polyphenols on T2D mice as well as the underlying mechanisms involved.

Insulin resistance is a typical characteristic of T2D. In this study, palmitic acid was used to induce an IR model in HepG2 cells to identify polyphenol compounds with hypoglycemic potential in BPE. Glucose consumption and glycogen levels are the most straightforward measures for evaluating the extent of insulin resistance in cells. The results indicated that after 24 h of BPE treatment, the glucose uptake ability and glycogen levels in IR cells were significantly improved, suggesting a marked improvement in the state of insulin resistance. Among the ten polyphenol monomers, dihydromyricetin, rutin, gallic acid, gentisic acid, syringic acid and catechol showed significant improvement in both indicators. Notably, dihydromyricetin remarkably enhanced the ability of cells to utilize glucose, while gallic acid exhibited consistently outstanding results in repeated validations. HK is the first key enzyme in the process of glucose breakdown, catalyzing the conversion of glucose to glucose-6-phosphate. PK catalyzes the terminal step of glycolysis and is one of the main rate-limiting enzymes in the glycolytic pathway. The decreased activities of HK and PK result in inadequate glucose utilization by liver cells during IR. G6P is a limiting enzyme in the gluconeogenesis process that hydrolyzes glucose-6-phosphate to generate glucose, contributing significantly to maintaining the dynamic balance of blood glucose [23]. In this study, BPE and some of its polyphenol compounds effectively alleviated the IR state in HepG2 cells and promoted glucose metabolism in IR-HepG2 cells, an effect achieved by influencing cellular glucose metabolism and the activity of key enzymes. Based on the above results, this study subsequently selected dihydromyricetin and gallic acid for further investigation.

IR refers to a diminished sensitivity to insulin in the body, characterized by hyperinsulinemia resulting from abnormal serum insulin levels [30]. An important metric for evaluating insulin resistance is the HOMA-IR, which is widely utilized in clinical practice [31]. A higher HOMA-IR score suggests more severe insulin resistance and reduced insulin sensitivity. This study demonstrated that treatment with BPE, along with polyphenol compounds, led to a notable reduction in FBG and fasting insulin levels in mice, resulting in marked improvements in insulin resistance. This suggests that blueberry polyphenols may improve insulin sensitivity and mitigate pancreatic β-cell damage associated with diabetes. Moreover, histological analysis of pancreatic tissue revealed that blueberry polyphenols provided a certain degree of protection to the damaged pancreatic islets, thereby ameliorating pancreatic lesions in diabetic mice.

The liver is crucial in glucose and lipid metabolism, serving as a central organ for maintaining metabolic homeostasis and is a significant target of systemic IR, thereby influencing the advancement of diabetes [32]. Patients with diabetes often exhibit elevated glycogenolysis and gluconeogenesis. Gluconeogenesis plays a crucial role in regulating normal blood glucose levels. Targeting hepatic gluconeogenesis to lower blood glucose levels represents a novel strategy for the management of T2D [33]. Hepatic glycogen acts as an essential glucose storage unit, and a reduction in glycogen synthesis and storage can disrupt glucose homeostasis, thereby adversely affecting blood glucose regulation. Glycogen synthesis is facilitated by glycogen synthase (GS), whose activity is negatively regulated by GSK3β. The enzymes PEPCK and G6Pase regulate hepatic gluconeogenic metabolism, serving as critical intermediates in the initiation of gluconeogenesis, which is regulated by FOXO1. PGC1α, a transcriptional coactivator, enhances Creb activity when overexpressed in the liver. Sik1 also functions to inhibit the expression of Creb [34]. Creb stimulates the expression of PEPCK and G6Pase, thereby modulating gluconeogenesis [35]. In this study, treatments with blueberry polyphenols and polyphenolic compounds have been demonstrated to inhibit hepatic glycogenolysis via reducing the activity of G6Pase and PEPCK, thereby modulating gluconeogenesis, reducing blood glucose levels in T2D mice and alleviating insulin resistance. This is consistent with previous studies [36].

**Figure 8:**
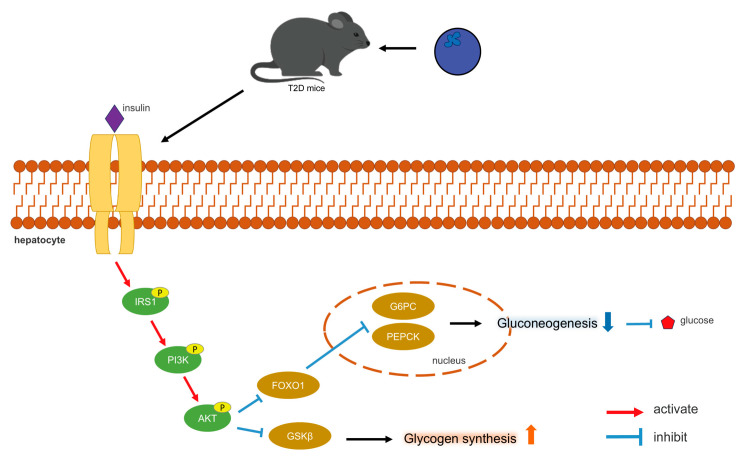
Mechanism of blood glucose regulation by blueberry polyphenols.

The PI3K/AKT pathway represents the primary pathway for insulin signaling, functioning as a central component in the regulation of glucose metabolic homeostasis and glycogen synthesis [37]. Insulin binds to receptors on the surfaces of hepatocyte membranes (Insulin receptor, INSR), leading to its phosphorylation, resulting in the activation of IRS-1. The phosphorylated IRS-1 subsequently interacts with the p85 subunit of PI3K, thereby activating downstream Akt and promoting insulin signaling. The stimulation of the PI3K/AKT pathway also facilitates the translocation of plasma glucose into hepatocyte cytoplasm, inducing glycogen synthesis [37]. Disruptions in the PI3K/AKT signaling pathway may result in IR, which further influences PI3K/AKT signaling, creating a vicious cycle [37]. The effect of insulin on hepatocytes is mediated by Akt activation [38]. The findings of this study indicated that blueberry polyphenols may activate the PI3K/AKT pathway, promote the phosphorylation of PI3K and AKT, thereby modulating insulin signaling and glucose metabolism. Subsequent to PI3K/AKT activation, GSK3β can be phosphorylated and inactivated, alleviating its inhibitory effect on GS [39]. FOXO1 is a particularly prominent AKT target, playing significant physiological roles in hepatocytes [40]. AKT inactivates FOXO1, thereby reducing the transcription levels of PEPCK and G6Pase, which subsequently decreases gluconeogenesis [41]. Figure 8 illustrates the mechanism by which blueberry polyphenols help lower blood glucose levels.

In diabetic patients, disturbances in glucose metabolism are frequently associated with disruptions in lipid metabolism [42,43]. Furthermore, dyslipidemia in diabetes can exacerbate insulin resistance. The main reason for lipid abnormalities in diabetes is reduced insulin sensitivity in adipose tissue and liver. HDL-C facilitates the transport of lipids from the vasculature to the liver for metabolic processing, while LDL-C carries lipids from liver to the bloodstream. TC and TG serve as indicators of lipid profile status. In this study, the intervention of BPE (along with polyphenolic components in BPE) led to varying degrees of alleviation in abnormal blood lipid levels in diabetic mice, suggesting that blueberry polyphenols can mitigate hyperlipidemia associated with metabolic disturbances in diabetes to some extent. Genes associated with lipid droplet proteins, such as *Cidea*, *Cidec*, *Plin4* and *Plin5*, are vital regulators of lipid deposition and the assembly of considerable lipid droplets in hepatocytes [44]. *Lpin1* and *Lpin2* work collaboratively to maintain triglyceride homeostasis [45]. Following the blueberry polyphenol intervention in this study, the expression of hepatic lipid droplet-associated genes was suppressed, resulting in a reduction of fat deposition and improved control of lipid metabolic disorders.

Oxidative stress is characterized by an imbalance between the generation of ROS and their removal during metabolic processes. Numerous studies indicate that oxidative stress plays a significant role in the advancement of diabetes [46,47]. Blueberry anthocyanins have been shown to reduce ROS levels in HepG2 cells stimulated by high glucose [48]. In this study, blueberry polyphenols diminished liver oxidative stress induced by hyperglycemia, which included increasing levels of the antioxidant enzyme catalase CAT and decreasing MDA levels, likely due to the antioxidant properties of polyphenols.

Certain questions remain significant for future research. For instance, in animal experiments, the administration of blueberry polyphenols and their polyphenolic components did not establish a clear concentration gradient to investigate whether the hypoglycemic effects of blueberry polyphenols exhibit a dose-dependent characteristics, which would provide essential dosage references for the subsequent use of blueberry polyphenols as dietary supplements. Additionally, numerous bioactive polyphenolic compounds are present in blueberry polyphenols; however, this study focuses solely on two of these compounds, thereby leaving the metabolic effects of other active components as important subjects for further investigation.

## 5. Conclusions

This study demonstrated that blueberry polyphenol extracts, along with two specific polyphenolic components (dihydroquercetin and gallic acid) present in BPE can modulate insulin signaling and glucose metabolism in diabetic mice by activating the PI3K/AKT pathway, thereby alleviating insulin resistance and ameliorating disorders of liver lipid metabolism. Corresponding changes in enzyme activity further suggested that blueberry polyphenols can reduce oxidative stress in the liver while exhibiting hepatoprotective properties. In summary, this research provides a solid foundation for the use of blueberry polyphenols as a dietary nutritional supplement for the prevention and management of diabetes.

## Figures and Tables

**Table 1:** Bioactive components of BPE.

Item	Content (mg/g)
Total polyphenols (as gallic acid)	635.58 ± 11.57
Total flavonoids (as rutin)	118.97 ± 1.98
Total anthocyanins (as cyanin-3-glucoside)	246.13 ± 3.80

Data were represented as mean ± SD (n = 4).

**Table 2:** Ten polyphenolic compounds in vitro.

Compound	Classification	Formula	CAS
Dihydromyricetin	flavanonol	C_15_ H_12_ O_8_	27200-12-0
Quercetin	flavonol	C_15_ H_10_ O_7_	117-39-5
Rutin	flavonol glycoside	C_27_ H_30_ O_16_	153-18-4
Chlorogenic acid	phenolic acid	C_16_ H_18_ O_9_	327-97-9
Gallic acid	phenolic acid	C_7_ H_6_ O_5_	149-91-7
Gentisic acid	phenolic acid	C_7_ H_6_ O_4_	490-79-9
Syringic acid	phenolic acid	C_9_ H_10_ O_5_	530-57-4
Methyl gallate	phenolic acid	C_8_ H_8_ O_5_	99-24-1
Pyrogallol	phenolic compound	C_6_ H_6_ O_3_	87-66-1
Catechol	phenolic compound	C_6_ H_6_ O_2_	120-80-9

## Data Availability

Data are contained within the article and Supplementary Materials.

## Supplementary Materials

The following supporting information can be downloaded at: https://www.mdpi.com/article/10.3390/antiox13121490/s1, Figure S1: Influence of blueberry polyphenols on cell viability in HepG2 cells, Table S1: Primers used for qPCR, Table S2: Polyphenolic compounds identified in BPE, Table S3: The ADMET parameters of dihydromyricetin and gallic acid via pkcsm tool (http://biosig.unimelb.edu.au/pkcsm/prediction).
