# Supplementary material for: Evaluation of Hypoglycemic Polyphenolic Compounds in Blueberry Extract: Functional Effects and Mechanisms
Source: Antioxidants (Basel). 2024 Dec 6;13(12):1490. doi: 10.3390/antiox13121490 (PMC11672983; doi:10.3390/antiox13121490)
Supplement: Supplementary file 1 [file antioxidants-13-01490-s001.zip › antioxidants-3313937-supplementary.pdf]

Supplementary Figures:

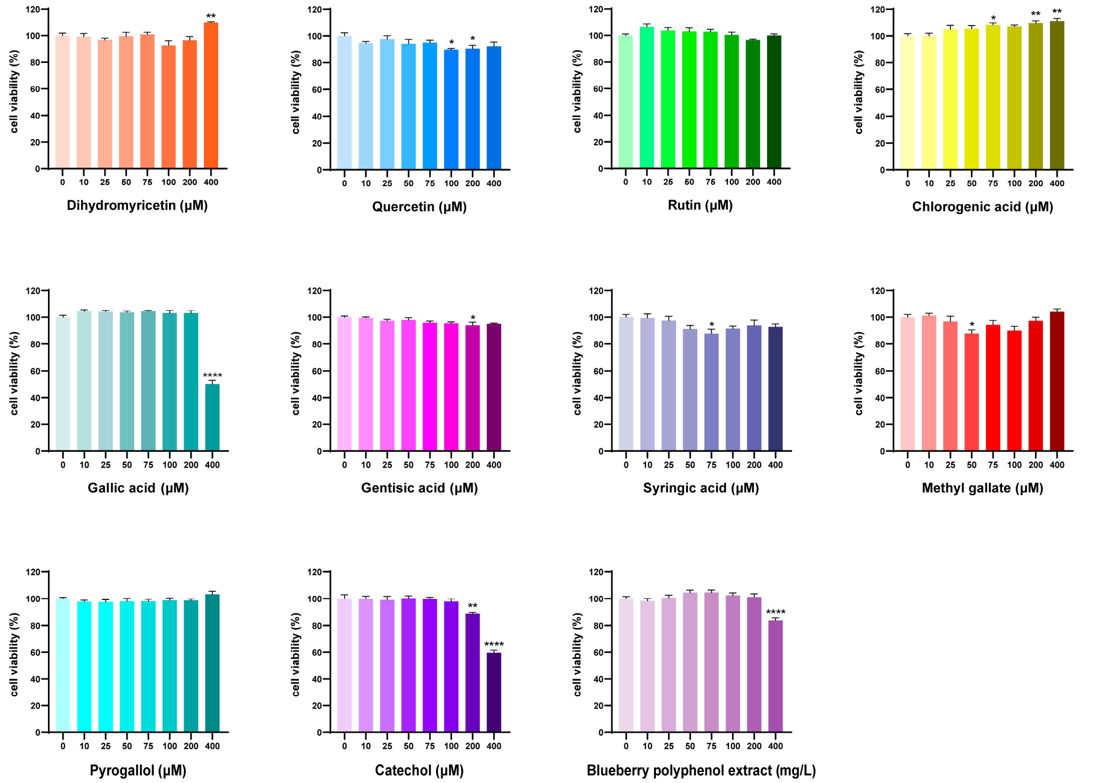

Figure S1. Influence of blueberry polyphenols on cell viability in HepG2 cells. Data are expressed as the mean ± SEM. *p<0.05 versus control group; **p<0.01 versus control group; ****p<0.0001 versus control group.

Supplementary Tables:

Table S1. Primers used for qPCR.

Primers	FORWARD (5′ to 3′)	REVERSE (5′ to 3′)	Accession number
Egfr	TTGGAATCAATTTTACACCGAAT	GTTCCCACACAGTGACACCA	NM_207655.2
Insr	CAAGAAATGATTCAGATGACAGCAG	AGACTCCATCCTTCAGGGACTCA	NM_001330056.1
IRS1	AGCAGCAGCAGCAGCAACAG	TCCTCCTTCTCCTCCTCCTCCTC	NM_010570.4
Pik3r5	AGCTTCCACACTACGGGTTG	GGTTCCGTGGCTTCTCTTCA	XM_006533548.3
AKT1	TTTGGGAAGGTGATTCTGGTG	CGTAAGGAAGGGATGCCTAGAGTT	NM_001409450.1
Gsk3β	TGGCAGCAAGGTAACCACAG	CGGTTCTTAAATCGCTTGTCCTG	XM_030249221.2
Gys2	CGCTCCTTGTCGGTGACATC	CATCGGCTGTCGTTTTGGC	NM_145572.3
Sik1	CTACAACCACTTTGCCGCCAT	AGGGGGAATAATAAGGGCTGAAG	XM_036160364.1
Creb	AGCAGCTCATGCAACATCATC	AGTCCTTACAGGAAGACTGAACT	XM_030245776.2
PGC1α	TATGGAGTGACATAGAGTGTGCT	CCACTTCAATCCACCCAGAAAG	NM_008904.3
FOXO1	TCAAGGATAAGGGCGACAGC	TGTCCATGGACGCAGCTCTT	NM_019739.3
Pck1	AAGGAGTGGAGACCGCAGGAC	TGCCGAAGTTGTAGCCGAAGAAG	NM_011044.3
G6PC	AGGTCGTGGCTGGAGTCTTGTC	AATGCAGGCGAAGCGGAATGG	NM_008061.4
GADPH	AATGTGTCCGTCGTGGATCTGA	AGTGTAGCCCAAGATGCCCTTC	NM_001411843.1

Table S2. Polyphenolic compounds identified in BPE.

Num	Name	Formula	Calc. MW	m/z	RT/min	Reference Ion	Group Area
1	Quercetin-3-β-D-glucoside	C21 H20 O12	464.09523	465.10236	18.371	[M+H]+1	26805713073
2	Reynoutrin	C20 H18 O11	434.08464	435.0918	18.775	[M+H]+1	10033393327
3	Cyanidin-3-O-glucoside chloride	C22 H24 O12	448.10021	449.10739	19.452	[M+H]+1	7587676337
4	Petunidin 3-O-galactoside	C22 H24 O12	480.12724	479.11996	20.124	[M-H]-1	5240915563
5	Quercitrin	C21 H20 O11	448.10081	447.09366	22.862	[M-H]-1	4020772162
6	Quercetin	C15 H10 O7	302.04222	303.04947	21.892	[M+H]+1	3621920264
7	3-Methoxy-5,7,3',4'-tetrahydroxy-flavone	C16 H12 O7	316.05739	317.06467	19.051	[M+H]+1	2328701732
8	Chlorogenic acid	C16 H18 O9	354.09522	353.08798	19.162	[M-H]-1	2158667437
9	D-(-)-Quinic acid	C7 H12 O6	192.06354	191.05626	19.161	[M-H]-1	1159152078
10	Avicularin	C20 H18 O11	434.08452	435.0918	22.67	[M+H]+1	959676207.9
11	Rutin	C27 H30 O16	610.15368	609.14661	21.543	[M-H]-1	887797336.3
12	Gallic acid	C7 H6 O5	170.0216	169.01436	7.99	[M-H]-1	697627927.2
13	Procyanidin B1	C30 H26 O12	578.14247	577.13562	18.517	[M-H]-1	692261684.5
14	7-Hydroxycoumarine	C9 H6 O3	162.03153	163.0388	19.164	[M+H]+1	654379676
15	Cyanidin	C15 H10 O6	286.04686	287.05414	18.837	[M+H]+1	465221753.6
16	Narcissoside	C28 H32 O16	624.16939	623.16241	22.449	[M-H]-1	429225829.5
17	Ethyl gallate	C9 H10 O5	184.03734	183.03006	18.042	[M-H]-1	376794096.3
18	Methyl gallate	C8 H8 O5	184.03734	183.03006	18.042	[M-H]-1	376794096.3
19	7-Methoxycoumarin	C10 H8 O3	176.04729	177.05457	22.433	[M+H]+1	345495956
20	Gentisic acid	C7 H6 O4	154.02674	153.01947	16.638	[M-H]-1	324442342.4
21	Syringic acid	C9 H10 O5	198.05271	199.05998	22.504	[M+H]+1	314597047.1
22	Delphinidin 3-O-galactoside	C21 H22 O12	466.11131	465.10403	21.533	[M-H]-1	266231680.9
23	Isorhamnetin	C16 H12 O7	316.05739	317.06467	22.886	[M+H]+1	244714103.5
24	Rhamnetin	C16 H12 O7	316.05739	317.06467	22.886	[M+H]+1	244714103.5
25	Dihydromyricetin	C15 H12 O8	320.0533	319.04602	19.754	[M-H]-1	242501878.2
26	Catechin	C15 H14 O6	290.07907	289.07199	19.132	[M-H]-1	232174223
27	Ferulic acid	C10 H10 O4	194.05776	195.06502	19.722	[M+H]+1	227641285
28	Pyrogallol	C6 H6 O3	126.03172	125.02444	7.987	[M-H]-1	209544340.8
29	Fisetin	C15 H10 O6	286.04686	287.05414	19.321	[M+H]+1	184298502.8
30	4-Hydroxybenzoic acid	C7 H6 O3	138.03175	183.02995	21.091	[M+FA-H]-1	157292016.3
31	4,5-Dicaffeoylquinic acid	C25 H24 O12	516.12715	515.11987	22.332	[M-H]-1	151784720.8
32	Myricetin	C15 H10 O8	318.03783	317.03055	20.584	[M-H]-1	140790859.1
33	4-Coumaric acid	C9 H8 O3	164.04742	163.04021	18.218	[M-H]-1	136516029.8
34	Diosmetin	C16 H12 O6	300.06246	301.06973	21.567	[M+H]+1	127316222
35	Astragalin	C21 H20 O11	448.10094	447.09366	23.911	[M-H]-1	110260901.1
36	Caffeic acid	C9 H8 O4	180.0424	179.03513	19.832	[M-H]-1	103386198.3
37	Catechol	C6 H6 O2	110.03678	109.0295	16.648	[M-H]-1	102570563.5
38	Kaempferol	C15 H10 O6	286.04686	287.05414	22.759	[M+H]+1	95970694.25
39	3,4-Dihydroxyphenylethanol	C8 H10 O3	154.06306	153.05579	17.493	[M-H]-1	93874362.66

40	3-Coumaric acid	C9 H8 O3	164.04748	163.04021	19.024	[M-H]-1	76698051.28
41	Kaempferol-3-O-rutinoside	C27 H30 O15	594.15898	593.15216	22.302	[M-H]-1	69077427.25
42	2,6-Dimethoxyphenol	C8 H10 O3	154.06291	155.07019	22.496	[M+H]+1	67518848.84
43	Arbutin	C12 H16 O7	272.08972	317.08789	6.003	[M+FA-H]-1	54583649.67
44	Isoferulic acid	C10 H10 O4	194.0581	195.06505	17.697	[M+H]+1	52694006.58
45	Isovanillic acid	C8 H8 O4	168.04215	169.04942	22.544	[M+H]+1	44684421.28
46	Vanillic acid	C8 H8 O4	168.04215	169.04942	22.544	[M+H]+1	44684421.28
47	Procyanidin A2	C30 H24 O12	576.12698	575.12024	21.352	[M-H]-1	37366957.45
48	Naringenin	C15 H12 O5	272.0685	271.06143	27.367	[M-H]-1	32674240.36
49	Eriodictyol	C15 H12 O6	288.06305	289.07019	23.169	[M+H]+1	31837767.98
50	Afzelin	C21 H20 O10	432.10599	431.09882	23.848	[M-H]-1	28846092.17
51	3,4-Dimethoxycinnamic acid	C11 H12 O4	208.07341	209.08069	21.564	[M+H]+1	28321821.49
52	3-Hydroxybenzoic acid	C7 H6 O3	138.03171	137.02443	16.527	[M-H]-1	23016458.08
53	Phloridzin	C21 H24 O10	436.13738	435.13013	23.389	[M-H]-1	20062860.44
54	Ethyl Paraben	C9 H10 O3	166.06294	167.07022	17.489	[M+H]+1	19470411.74
55	Salicylic acid	C7H6O3	168.04234	167.03506	17.377	[M-H]-1	16825148.86
56	5-Methoxysalicylic acid	C8 H8 O4	168.04234	167.03506	17.377	[M-H]-1	16825148.86
57	Ethyl caffeate	C11 H12 O4	208.07368	207.06641	26.16	[M-H]-1	9449217.718

Table S3. The ADMET parameters of dihydromyricetin and gallic acid via pkcsm software.

	Predicted Value	Dihydromyricetin	Gallic acid
Absorption			
	Water solubility (log mol/L)	-2.974	-2.56
	Caco2 permeability (log Papp in 10-6 cm/s)	0.111	-0.081
	Intestinal absorption (human) (% Absorbed)	58.92	43.374
	Skin Permeability (log Kp)	-2.735	-2.735
	P-glycoprotein substrate	Yes	No
	P-glycoprotein I inhibitor	No	No
	P-glycoprotein II inhibitor	No	No
Distribution			
	VDss (human) (log L/kg)	1.662	-1.855
	Fraction unbound (human) (Fu)	0.39	0.617
	BBB permeability (log BB)	-1.167	-1.102
	CNS permeability (log PS)	-3.586	-3.74
Metabolism			
	CYP2D6 substrate	No	No
	CYP3A4 substrate	No	No
	CYP1A2 inhibitior	No	No
	CYP2C19 inhibitior	No	No
	CYP2C9 inhibitior	No	No
	CYP2D6 inhibitior	No	No
	CYP3A4 inhibitior	No	No
Excretion			
	Total Clearance (log ml/min/kg)	0.278	0.518
	Renal OCT2 substrate	No	No
Toxicity			
	AMES toxicity	No	No
	Max. tolerated dose (human) (log mg/kg/day)	0.4	0.7
	hERG I inhibitor	No	No
	hERG II inhibitor	No	No
	Oral Rat Acute Toxicity (LD50) (mol/kg)	2.433	2.218
	Oral Rat Chronic Toxicity (LOAEL) (log mg/kg_bw/day)	3.152	3.06
	Hepatotoxicity	No	No
	Skin Sensitisation	No	No
	T.Pyriformis toxicity (log ug/L)	0.285	0.285
	Minnow toxicity (log mM)	6.479	3.188
